# Complete Genome Sequence of Nova Virus, a Hantavirus Circulating in the European Mole in Belgium

**DOI:** 10.1128/genomeA.00770-15

**Published:** 2015-08-06

**Authors:** Lies Laenen, Valentijn Vergote, Inne Nauwelaers, Ina Verbeeck, Liana E. Kafetzopoulou, Marc Van Ranst, Piet Maes

**Affiliations:** Laboratory of Clinical Virology, Rega Institute for Medical Research, KU Leuven, Leuven, Belgium

## Abstract

The complete genome sequence of Nova virus, a divergent hantavirus, originating from the kidney tissue of a European mole (*Talpa europaea*) from Belgium was determined. The 3 genomic segments have a total length of 11,979 nucleotides, and nucleotide identities to other Nova viruses are between 80 and 89%.

## GENOME ANNOUNCEMENT

Hantaviruses are zoonotic viruses that can cause hemorrhagic fever with renal syndrome or hantavirus pulmonary syndrome upon human infection. In accordance with other members of the *Bunyaviridae* family, hantaviruses are enveloped negative-sense single-stranded RNA viruses with a trisegmented genome (1). Although hantaviruses were previously presumed to be primarily rodent-borne viruses, the recognized host range has expanded as a result of the detection of multiple hantaviruses in shrews, moles, and bats over the last decade (2–5). These discoveries led to the hypothesis of a complex evolutionary history with multiple host switching events where ancestors of insectivores rather than rodents appear to be the natural hosts of primordial hantaviruses (6). While the number of identified insectivore-borne hantaviruses is vastly expanding, little is known about their pathogenicity, and the available sequence information remains limited (7). In 2009, Nova virus (NVAV), a highly divergent hantavirus, was detected in the archival liver tissue of the European mole (*Talpa europaea*) in Zala, Hungary (8). Here, we report the first complete genome characterization of a Nova virus from a European mole originating from Belgium.

Kidney tissue was obtained from a wild-caught European mole in 2013 in Namur, Belgium, and transferred to RNA*later* stabilization solution (Life Technologies). Total RNA was extracted from kidney homogenate with the RNeasy minikit (Qiagen), and viral RNA was amplified using the OneStep RT-PCR kit (Qiagen). Primers for conserved regions were designed based upon alignments of available Nova virus sequences or other insectivore-borne hantaviruses when insufficient sequence data were present. PCR amplicons were purified using ExoSAP-IT (Affymetrix) and sequenced with the BigDye Terminator v3.1 cycle sequencing kit (Life Technologies) on an Applied Biosystems 3130xl Genetic Analyzer.

The genome of this strain, designated NVAV BE/Namur/TE/2013/1, has a full length of 11,979 nucleotides with a G+C content of 35.65%. Complete genomic characterization reveals a 1,826-nt S segment (encoding a 428-amino-acid (aa) nucleocapsid protein, but lacking a secondary open reading frame for the NSs protein), a 3,590-nt M segment (encoding a 1,127-aa glycoprotein precursor), and a 6,563-nt L segment (encoding a 2,157-aa polymerase gene). Nucleotide similarity to other Nova viruses ranged from 80 to 89%, 82 to 86%, and 82 to 88% for S, M, and L segments, respectively. Amino acid similarity to other Nova viruses ranged between 97 and 98% (nucleocapsid), 98% (glycoprotein precursor), and 96 to 97% (polymerase). Phylogenetic analyses of full-length S, M, and L segments demonstrated a host-specific clustering with other Nova viruses. Nova virus exhibited a close phylogenetic relationship with shrew- and bat-borne hantaviruses rather than other mole-borne hantaviruses, providing arguments for a complex hantavirus evolution likely involving host switching events.

### Nucleotide sequence accession numbers.

The complete genome of Nova virus strain BE/Namur/TE/2013/1 has been deposited in GenBank under the accession numbers KT004445, KT004446, and KT004447.
